# Evaluation of service-user and clinician feedback of ‘Beth’: a new digital tool in South London and Maudsley NHS Foundation Trust

**DOI:** 10.1192/j.eurpsy.2023.396

**Published:** 2023-07-19

**Authors:** D. Ragupathy, B. Arroyo, G. Gillett

**Affiliations:** ^1^ King’s College London; ^2^ South London and Maudsley NHS Foundation Trust; ^3^Institute of Psychiatry, Psychology & Neuroscience, King’s College London, London, United Kingdom

## Abstract

**Introduction:**

Conventional healthcare records are generally inaccessible to service-users. ‘Beth’ is a digital tool in South London and Maudsley NHS Foundation Trust which allows service-users to self-monitor symptoms, set therapeutic goals, access aspects of clinical records and communicate with care teams.

**Objectives:**

To explore service-user and clinician perspectives of Beth, and to understand how Beth might impact clinical care.

**Methods:**

Service-user and clinician users completed an online questionnaire. Likert-scale and free-text response questions covered user experience, impact on clinical care and suggested improvements. N=26 service-users and 43 clinicians completed the questionnaire. Quantitative and qualitative analyses are presented.

**Results:**

Service-users reported the most useful features were tracking sleep and mood, messaging their care team, logging coping strategies and viewing care plans, goals and upcoming appointments. A majority reported Beth improved clinical assessments and they would recommend it to others. Barriers to using Beth included navigational difficulties, lack of access to internet or hardware, needing to register for an account and forgetting to use it. Clinicians reported booking appointments, messaging service-users, sharing care plans and accessing mood diaries were the most useful features. However, many clinicians did not use Beth regularly. Barriers included difficulties using Beth, finding it time-consuming and reportedly poor service-user adherence.

**Conclusions:**

Our findings highlight potential benefits of digital tools in mental health care, alongside numerous barriers and suggested improvements. Limitations include a small sample size and lack of objective user data. Future work may involve qualitative interviews, analysis of objective usage data and trialing improvements in service design.

**Disclosure of Interest:**

None Declared

